# Prevalence, risk factors and molecular identification of paramphistomid species in sheep from a Spanish endemic area

**DOI:** 10.1186/s13620-024-00283-y

**Published:** 2024-11-26

**Authors:** David García-Dios, Pablo Díaz, Susana Remesar, Miguel Viña, Néstor Martínez-Calabuig, Ana Saldaña, Pablo Díez-Baños, Rosario Panadero, Patrocinio Morrondo, Ceferino Manuel López

**Affiliations:** 1 Faculty of Veterinary Sciences, Universidade de Santiago de Compostela, Investigación en Sanidad Animal: Galicia (Grupo INVESAGA), Lugo, Spain; 2 IBADER- Instituto de Biodiversidade Agraria e Desenvolvemento Rural, Lugo, Spain

**Keywords:** Paramphistomids, *Calicophoron daubneyi*, Risk factors, Molecular identification, Sheep, Roe deer, Spain

## Abstract

**Background:**

Paramphistomids are ruminal trematodes that cause important losses in tropical and subtropical regions. However, their presence in Europe has increased significantly in recent decades. In northwestern Spain, this trend has been confirmed in cattle, but data in sheep are scarce and not updated. Moreover, the paramphistomid species affecting sheep in the area have never been molecularly identified. To evaluate the prevalence of paramphistomid infections in sheep from northwestern Spain, 826 faecal samples from 25 sheep farms were collected and analysed via coproscopic techniques. In addition, the rumens of 85 roe deer from the same area were examined to detect adult paramphistomids. The species present were molecularly identified. Multivariate analyses for identifying the risk factors affecting the prevalence and egg shedding of rumen flukes were also performed.

**Results:**

Overall, 14% of the animals and 44% of the flocks were positive; the mean egg count was 20.5 eggs per gram of faeces. In contrast, no adult paramphistomids were found in roe deer. Older sheep, those from farms located in the central climatic area, without water throughs available on pastures and using their own manure to fertilize, were considered significantly more susceptible to infection with paramphistomids. With respect to egg shedding, animals from 37 to 72 months of age, farms under semiextensive management, and those coinfected with *Fasciola hepatica* presented significantly greater egg counts. Molecular identification revealed 100% similarity with *Calicophoron daubneyi* sequences from other European and Mediterranean countries.

**Conclusions:**

The present study confirms the increase in the prevalence of paramphistomid infections in sheep in the area with high dissemination of the parasite, as previously reported in cattle, and represents the first molecular identification of *C. daubneyi* in sheep from Spain. Our results demonstrate that special attention should be given to adult animals since they are the main carriers and are responsible for environmental contamination. In addition, detecting risk areas and applying effective control management measures such as the installation of watering points on pastures seems essential for limiting infections in livestock, especially in sheep, since they are susceptible to developing clinical paramphistomidosis at any time in their lives. The absence of adult flukes in roe deer suggests that they represent less suitable hosts for this trematode than cattle and sheep, although more robust studies monitoring the situation in sympatric areas with domestic ruminants are needed.

**Supplementary Information:**

The online version contains supplementary material available at 10.1186/s13620-024-00283-y.

## Background

Paramphistomids are ruminal trematodes distributed worldwide that cause significant economic losses, especially in tropical and subtropical areas, where they are particularly common [1–3]. Rumen flukes have an indirect life cycle; mud or freshwater snails act as intermediate hosts, and ruminants act as definitive hosts [4]. After ingesting metacercariae when grazing, juvenile flukes start feeding on the duodenal mucosa [2, 5]; intense infections with immature flukes can lead to clinical gastrointestinal signs such as liquid or haemorrhagic diarrhoea, weakness and apathy, submandibular oedema, anaemia, or dehydration [6, 7]. Clinical cases are especially common in both cattle and small ruminants in their first grazing season; however, sheep and goats of any age can show clinical paramphistomidosis [6]. Although adult paramphistomids are considered well tolerated by the host, extensive lesions and inflammation at the attachment sites of the parasites have been reported [8]; therefore, their negative impact on productions must be further assessed.

Prior to the 1990s, rumen flukes, typically identified as *Paramphistomum leydeni* or *Paramphistomum cervi*, were occasionally detected in ruminants from different European countries [9]. However, a marked increase in their prevalence was observed in cattle from central France during the 1990s [10]. Since then, a similar trend has been reported in cattle from several European countries, such as Belgium [11], the Czech Republic [12], Ireland [13], Italy [14], the Netherlands [15], and the United Kingdom [16]. The same trend was also observed in goats from France [17], sheep from Ireland, Scotland, Germany and Italy [18–21] and cervids from Ireland [22], although with a lower prevalence than in cattle. In Spain, the prevalence of rumen flukes is usually greater in cattle from the north of the country (12-33.9%) [1, 23–28] than in those from central areas (6.2%) [29] because climatic conditions favour the parasite life cycle. Prevalence data from different decades are only available for cattle from Galicia, Spain’s most northwestern region, which also demonstrated an increasing trend [1, 23–26, 28]. In contrast, information on other ruminants from Spain is very limited, with a high prevalence (50%) in red deer from Salamanca [30] and a low prevalence in sheep (0.7%) and goats (0.8%) from Galicia [31, 32]. Notably, no roe deer were found to be positive for rumen flukes in Spain [1].

The available data provide strong evidence that *Calicophoron daubneyi* is the major paramphistomid species in ruminants from Europe [4], although *P. leydeni* has been occasionally identified in sheep, red deer, and fallow deer from Ireland [21, 33], roe deer from Romania [34] and cattle from Germany and Austria [35]. Nevertheless, the molecular identification of paramphistomids in Spain has been performed only on samples recovered from cattle, allowing the detection of only *C. daubneyi* [1, 29].

Considering the scarcity of information on the presence of paramphistomid species in ruminants other than cattle from Europe, especially from Spain, the present study aimed to provide up-to-date and robust data on the prevalence of rumen fluke infections in sheep and roe deer from Galicia. In addition, a risk analysis was performed to identify those factors that significantly influence the probability of infection and egg shedding. Finally, a molecular was performed for identifying the predominant rumen fluke species infecting sheep from this area.

## Methods

### Study area

The study area is located in northwestern Spain (41^o^ 49’ − 43^o^ 47’ N and 6^o^ 42’ − 9^o^ 18’W), a region with a markedly oceanic climate, characterised by moderate temperatures and high rainfall [36], where three geoclimatic zones have been recorded [37] (Supplementary Fig. 1). Sheep flocks in the study area are generally small (mean number of animals per flock = 9) and belong to nonprofessional lamb-producing farms [38]. Sheep are usually reared in a semiextensive system, grazing during the day and kept indoors at night; sharing pastures with other ruminants, such as goats or cattle, is also common in this region.

### Sample and data collection

**Fig. 1:**
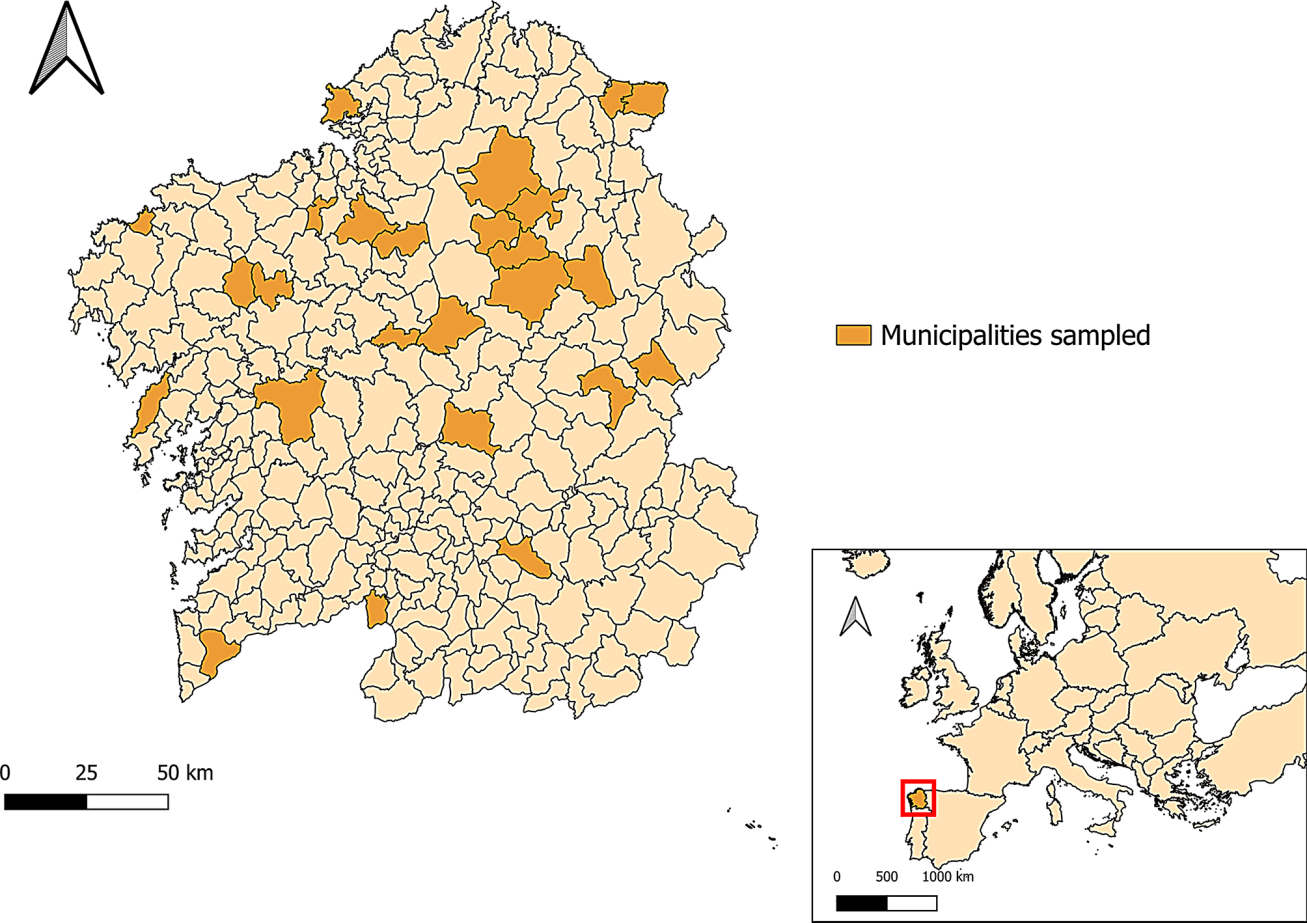
Location of the sampled sheep flocks in northwestern Spain

The number of farms needed for performing this study was calculated using the n.for.survey function included in the epiDisplay R statistical package [39] considering a 95% confidence interval, a precision of 80% and a prevalence of 50%, leading to the highest sampling. Thus, the minimum required number of farms was 24; finally, 25 flocks located in different municipalities were visited (Fig. 1). The number of samples collected from each farm was calculated considering a 95% confidence interval, with a precision of 90% and a prevalence of 50%. A total of 826 stool samples were collected between November 2020 and September 2022. All the samples were collected directly from the rectum of the animals, kept at 4 °C and individually analysed within 24 h.

At the time of sample collection, relevant information was also gathered by conducting an epidemiological survey. In this survey the age of the animals as well as data on the geographical location and general and sanitary management of the farms were recorded (Table 1).

In parallel with the sampling at sheep farms, the rumen of 85 roe deer from the province of Lugo, an area of Galicia densely populated by these wild ruminants, was thoroughly examined to detect adult paramphistomids. These animals originated from a wildlife recovery centre and were sent to the laboratory of the INVESAGA Group at the Faculty of Veterinary Medicine in Lugo.

### Parasitological techniques

Faecal samples were first analysed using a sedimentation quantitative technique for detecting trematode eggs [40]; the detection limit of this technique was 1 egg per gram of faeces (epg). In addition, the modified McMaster method using saturated saline solution was performed [41] to identify other parasitic forms, such as gastrointestinal nematode and cestode eggs or coccidian oocysts. The detection limit of this technique was 50 eggs/oocysts per gram of faeces (epg/opg).

### Statistical analysis

A mixed logistic regression was performed to identify those factors significantly affecting the probability of infection by paramphistomids (Table 1). The farm was included as a random variable. Factors were manually removed stepwise forward and backwards on the basis of the Akaike information criterion (AIC) value until the best model was obtained. Odds ratios were calculated by raising the constant *e* to the obtained estimators. This analysis was performed using the glmer() function of the lme4 package [42] in the R statistical package (R v.4.2.2; R Core Team, 2022). The possible influence of the variables summarized in Table 1 on paramphistomid egg shedding was assessed using a multivariate ANOVA; only positive animals (*n* = 115) were included, and the logarithm of paramphistomid egg shedding was used as the dependent variable. The step() function was applied to the initial model, and factors were eliminated step by step forward and backwards on the basis of the AIC value until the best model was obtained. Pairwise analyses were performed on ANOVA with the TukeyHSD() function.

### Molecular analysis

The sediment of all the positive samples from seven out of the eleven positive farms was pooled obtaining a single pool for each farm. DNA of each pool was extracted using a commercial kit (QIAamp Fast DNA Stool Mini Kit, Quiagen N.V.^®^, Venlo, The Netherlands) following the manufacturer’s instructions. The DNA samples were stored at -20 °C until analysis. All samples were first tested using a PCR protocol for amplification of an ≈ 500-bp fragment of the internal transcribed spacer 2 (ITS-2) of Trematoda using previously reported primers (ITS-2Trem F: TGTGTCGATGAAGAGCGCAG and ITS-2Trem R: TGGTTAGTTTCTTTTCCTCCGC) and protocols [43]. DNA of *C. daubneyi* obtained from a pool of cattle faeces, and nuclease-free water were included as positive and negative controls, respectively. After detecting nonspecific reactions, a new set of primers for detecting the partial ITS-2 region of Trematoda in faecal samples was designed. Partial nucleotide sequences of different Trematoda at this target (Supplementary Table 1) were used for designing the primers; these sequences were first aligned using the online tool GenomeNet Multiple Sequence Alignment by CLUSTALW (https://www.genome.jp/tools-bin/clustalw). Finally, a primer set (Table 1) was designed using the online software Primer-BLAST (https://www.ncbi.nlm.nih.gov/tools/primer-blast/index.cgi?GROUP_TARGET=on). The quality of the obtained primers was checked using the online tool Multiple Primer Analyser (Thermo Fisher Scientific) (https://www.thermofisher.com/es/es/home/brands/thermo-scientific/molecular-biology/molecular-biology-learning-center/molecular-biology-resource-library/thermo-scientific-web-tools/multiple-primer-analyzer.html); the results are summarized in Supplementary Table 2.

The amplification mixture for PCR contained 2 mM MgCl2, 200 µM dNTPs, 0.5 µM each primer and 0.5 units of NZYTaq II DNA polymerase (NZYTech, Lisbon, Portugal) in a final volume of 25 µl. Amplification was carried out in a T100 Thermal Cycler Bio-Rad (Hercules, California, USA), and the cycling conditions are summarized in Table 1. All PCR-positive samples, including the positive control, were purified and sequenced in both senses on an ABI 3730xl (Applied Biosystems, Foster City, California, USA) using a Big Dye Terminator v3.1 cycle sequencing kit (Applied Biosystems, Foster City, California, USA) at the Sequencing and Fragment Analysis Unit of the Santiago de Compostela University (Spain). The sequences were aligned and edited using ChromasPro (Technelysium, Brisbane, Australia), and the consensus sequences were scanned against the GenBank database using the Basic Local Alignment Search Tool (BLAST; http://blast.ncbi.nlm.nih.gov/Blast.cgi).

**Table 1:** Primer set and conditions of the novel PCR targeting a partial region of the internal transcribed spacer 2 of Trematoda

Primer	Label	Thermal profile			
		Pre-denaturation	Cyclic denaturation, annealing and extension	No. of cycles	Extension
ACTGCATACTGCTTTGAACAT	ITS2_TremF	95 °C / 10 min.	94 °C / 30 s.	35	72 °C / 10 min.
AAGTTCAGCGGGTATTCACG	ITS2_TremR		58 °C / 30 s.		
			72 °C / 1 min.		

### Phylogenetic analysis

A phylogenetic analysis was carried out using MrBayes 3.2.7 software [44] by Bayesian approach with Markov Chain Monte Carlo sampling (10,000,000 generations sampling every 1,000 steps). A Hasegawa-Kishino-Yano substitution model with gamma-distributed rate variation across sites (HKY + G) was used for the analysis of the sequences in the ITS-2 region. The model was selected on the basis of the AIC value using the free software jModelTest v.2.1.10 [45]. The tree was visualized and edited using FigTree 1.4.3 (http://tree.bio.ed.ac.uk/software/figtree/).

## Results

### Prevalence and egg shedding

**Fig. 2:**
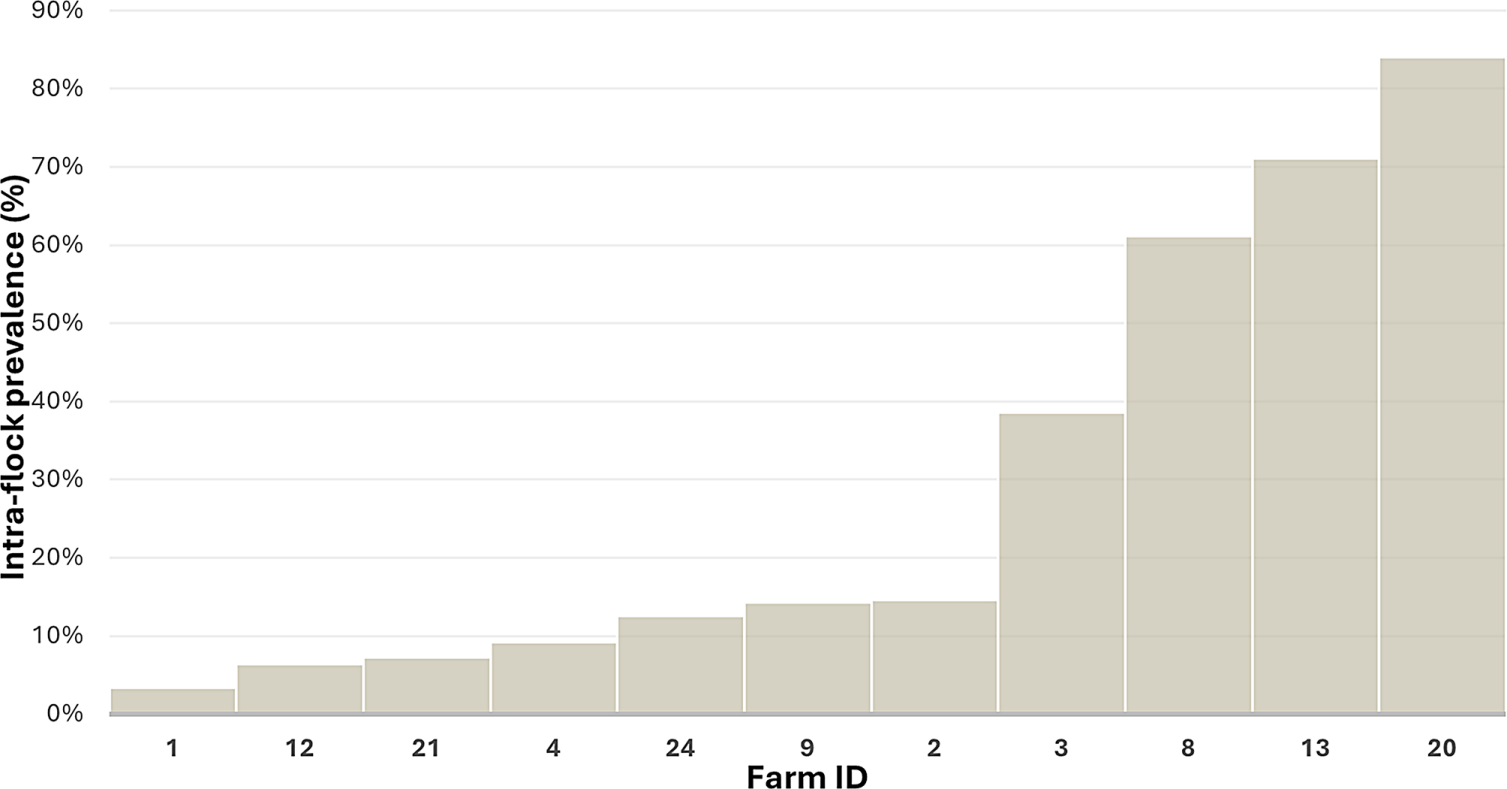
Paramphistomid intraflock prevalence in sheep farms from northwestern Spain

A total of 14% (95% CI 11.7–16.6) of the sheep sampled were positive, with a mean egg count of 20.5 epg (Standard deviation (SD) 37.4; range 1–202 epg). Moreover, 75% of the positive animals shed less than 20 epg; in addition, counts above 50 epg were not common since they were detected in 13.9% of the positive animals. No adult paramphistomids were found in any of the 85 roe deer rumens examined. A noticeable percentage of the flocks (44%; 95% CI 25.0–64.7) showed at least one animal shedding rumen fluke eggs, with a mean intraflock prevalence of 25.7% (95% CI 21.8–30.0). In most farms (63.6%), fewer than 20% of the animals shed paramphistomid eggs; in contrast, high intraflock prevalence rates (> 50%) were found in 27.3% of the farms (Fig. 2).

### Risk factor analysis

A total of 26 independent variables were extracted from the epidemiological survey. The prevalence and egg output values ranged from 0 to 61.1% and from 0 to 68.18 epg, respectively (Table 2).

**Table 2:** Variables and categories considered in the risk factor analysis affecting the prevalence and egg output of paramphistomids in sheep from northwestern Spain

Variable	Categories	Positive animals/total (%; CI 95%)	Mean egg output (SD)
Geoclimatic area	Centre	100/404 (24.8%; 20.7–29.3)	22.06 (39.14)
	Coast	1/183 (0.6%; 0.03–3.5)	9.29 (18.44)
	Mountain	14/237 (5.9%; 3.4–9.9)	2 (0)
Age (months)	1–12	5/107 (4.7%; 1.7–11.1)	1.9 (0.89)
	13–36	32/283 (11.3%; 8-15.3)	12 (20.59)
	37–72	43/268 (16%; 12-21.2)	31.21 (51.26)
	> 72	33/154 (21.4%; 15.4–28.9)	18.77 (28.68)
Management	Extensive	27/138 (19.6%; 13.5–27.4)	5.67 (9.56)
	Semiextensive	88/686 (12.8%; 10.5–15.6)	25.06 (41.45)
Flock size	6–82 sheep	34/235 (14.5%; 10.4–19.8)	34.77 (49.72)
	> 82 sheep	81/589 (13.8%; 11.1–16.9)	14.52 (29.2)
Other animals in the farm	No	1/115 (0.9%; 0.05–5.5)	2
	Ruminants	42/328 (12.8%; 9.5–17)	7.64 (13.44)
	Other	72/381 (18.9%; 15.2–23.3)	27.9 (44.3)
Introduction of external animals	No	39/227 (17.2%; 12.6–22.9)	31.59 (47.24)
	Only males	32/248 (12.9%; 9.1–17.9)	5.13 (8.86)
	Males and females	44/349 (12.6%; 9.4–16.7)	21.86 (37.34)
Sharing pastures	No	73/511 (14.3%; 11.4–17.7)	27.9 (44.3)
	Yes	42/313 (13.4%; 9.9–17.8)	7.64 (13.44)
Proximity of sheep farms	No	69/313 (22%; 17.7–27.1)	29.39 (45.13)
	Yes	46/511 (9%; 6.7–11.9)	7.17 (12.94)
Proximity of cattle farms	No	36/123 (29.3%; 21.6–38.2)	36.22 (40.06)
	Yes	79/701 (11.3%; 9.1–13.9)	17.9 (36.12)
Anthelmintics used	Benzimidazoles	27/100 (27%; 18.8–37)	5.67 (9.56)
	Macrocyclic lactones	28/229 (12.2%; 8.4–17.4)	36.32 (53.76)
	Benzimidazoles + Macrocyclic lactones	49/477 (10.3%; 7.8–13.4)	19.86 (21.32)
	Oxyclozanide + Benzimidazoles	11/18 (61.1%; 36.1–81.7)	19.86 (21.32)
Treatment frequency	Once a year	0/15 (0%; 0-25.3%)	0
	Spring and autumn	115/749 (15.4%; 12.9–18.2)	20.5 (37.43)
	Every 3 months	0/60 (0%; 0-7.5)	0
Quarantine	No	45/411 (11%; 8.2–14.5)	29 (45.34)
	Yes	70/413 (17%; 13.5–21)	15.04 (30.42)
Type of bedding	Out (extensive)	0/68 (0%; 0-6.7)	0
	Straw	115/756 (15.2%; 12.8–18)	20.5 (37.429)
Type of floor	Cement	61/349 (17.5%; 13.7–22)	20.39 (40.29)
	Out (Extensive management)	0/68 (0%; 0-6.7)	0
	Straw	54/407 (13.3%; 10.2–17)	20.63 (34.26)
Watering points cleaning frequency	Not applicable	1/104 (1%; 0.05-6)	1
	Each 1–7 days	72/272 (26.5%; 21.4–32.2)	16.81 (30.73
	Depending on usage	42/448 (9.4%; 6.9–12.6)	27.31 (46.66)
Bedding cleaning frequency	Weekly	14/169 (8.3%; 4.8–13.8)	15.86 (20.1)
	Monthly	42/290 (14.5%; 10.7–19.2)	23.69 (37.34)
	Yearly	58/289 (20%; 15.7–25.3)	19.34 (40.51)
	Not applicable (extensive management)	1/76 (1.3%; 0.07–8.1)	1
Corridors cleaning frequency	Daily	6/175 (3,4%; 1.4–7.7)	18.33 (26.58)
	Weekly	27/115 (23.5%; 16.3–32.5)	5.67 (9.56)
	Not applicable	82/534 (15.4%; 12.5–18.8)	25.55 (42.41)
Pasture rotation	No	12/49 (32.4%; 18.6–49.9)	18 (21.02)
	Yes	103/675 (13.3%; 11-15.9)	20.8 (38.94)
Presence of water courses or wetlands	No	7/110 (6.4%; 2.8–13.1)	2.43 (2.15)
	Yes	108/714 (15.1%; 12.6–18)	21.68 (38.33)
Presence of watering points in pastures	No	80/474 (16.9%; 13.7–20.6)	26.14 (42.77)
	Yes	35/350 (10%; 7.2–13.8)	7.63 (14.11)
Manure used as fertilizer	Own	107/487 (22%; 18.4–26)	21.52 (38.53)
	Other ruminant farms	2/101 (2%; 0.3–7.7)	2 (1.41)
	Others	6/236 (2.5%; 1-5.7)	8.5 (11.02)
Young and adult animals grazing together	No	0/60 (0%; 0-7.5)	0
	Yes	115/764 (15%; 12.6–17.8)	20.5 (37.43)
Age at which young animals are released to pasture	Immediate	65/251 (25.9%; 20.7–31.9)	31.05 (46)
	After colostrum feeding	3/85 (3.5%; 0.9–10.7)	2.33 (1.53)
	1–3 months	4/158 (2.5%; 0.8–6.8)	26.5 (30.16)
	> 3 months	43/270 (15.9%; 11.9–21)	5.28 (8.69)
Positivity to coccidia	No	16/146 (11%; 6.6–17.6)	18.56 (25.99)
	Yes	99/675 (14.7%; 12.1–17.6)	20.82 (39.05)
Positivity to gastrointestinal strongyles	No	48/206 (23.3%; 17.8–29.8)	23.44 (43.94)
	Yes	67/615 (10.9%; 8.6–13.7)	18.4 (32.14)
Positivity to *Fasciola hepatica*	No	97/802 (12%; 10-14.6)	12.35 (18.68)
	Yes	14/21 (66.7%; 43,1-84.5)	68.18 (71.01)
Season	Spring	4/179 (2.2%; 0.72-6)	2.25 (1.26)
	Summer	5/202 (2.5%; 0.91-6)	21.4 (28.5)
	Autumn	30/133 (22.6%; 15.96–30.8)	32.83 (53.07)
	Winter	76/310 (24.5; 19.9–29.8)	16.54 (30.03)

CI95%: confidence interval 95%; SD: standard deviation

The statistical analysis revealed that four factors significantly influence the probability of infection by paramphistomids: age, climatic area, presence of water troughs in the pasture, and the origin of the manure used on the pasture (Table 3). The prevalence increased progressively with age, and the probability of being infected was significantly lower in the youngest animals (Table 3). In addition, those animals over 72 months of age were 2.3 times more likely to be infected than those aged 13–36 months. With respect to the climatic area, sheep from the central plateau area had 9.4- and 83-fold greater probability of infection by paramphistomids than sheep from mountainous and coastal areas, respectively. The probability of infection was also 16.4 times greater among sheep from farms without water troughs available on pasture. Finally, sheep from farms that use their own manure to fertilize pastures were 568.2 times more likely to be infected than sheep from farms that use manure from animals other than ruminants and up to 798.39 times more likely than sheep from farms that use manure from external ruminants.

**Table 3:** Model obtained by mixed logistic regression for the prevalence of paramphistomids in sheep

Factor	Estimate	Z value	Probability (*P*)	OR	CI 95%
Age 1 (1–12 months)	-	-	-	-	-
Age 2 (13–36 months)	1.939	3.052	0.002	6.9	2-24.1
Age 3 (37–72 months)	2.057	3.245	0.001	7.8	2.3–27.1
Age 4 (> 72 months)	2.754	4.061	< 0.001	15.7	4.2–59.3
Climatic Area Centre	-	-	-	-	-
Climatic Area Coast	-4.415	-3.608	< 0.001	0.01	0.001–0.13
Climatic Area Mountain	-2.242	-3.099	0.002	0.11	0.03–0.44
Water troughs NO	-	-	-	-	-
Water troughs YES	-2.796	-3.844	< 0.001	0.06	0.01–0.25
Own manure	-	-	-	-	-
External ruminant manure	-4.703	-4.033	< 0.001	0.01	0.001–0.09
Other origin manure	-4.549	-4.624	< 0.001	0.01	0.002–0.07

*The hyphen (-) represents the reference category of each variable

**Fig. 3:**
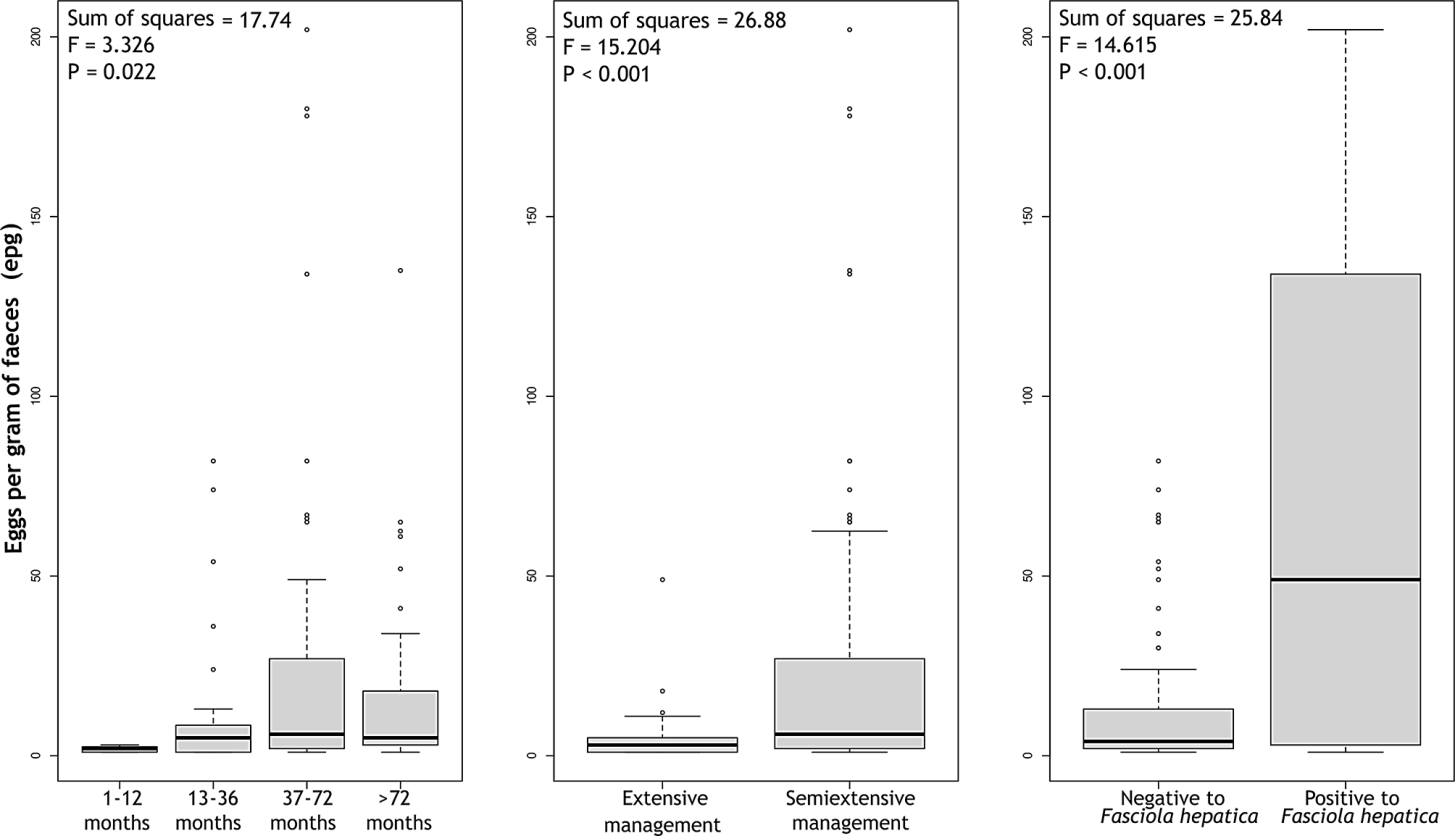
Box plot representing the distribution of paramphistomid egg shedding according to age, type of management and infection with *F. hepatica*

Finally, three variables were identified as significantly influencing paramphistomid egg counts: age, type of management, and infection with *F. hepatica* (Fig. 3). The Tukey HSD test revealed that, with respect to age, there were significant differences only between the groups of animals aged 1 to 12 months and those aged 37 to 72 months, with the latter exhibiting the greatest egg shedding. Semiextensive farms also had significantly greater egg counts than extensive farms, and animals positive for *F. hepatica* had significantly greater egg shedding than negative animals (Fig. 3).

### Molecular identification of paramphistomid species

**Fig. 4:**
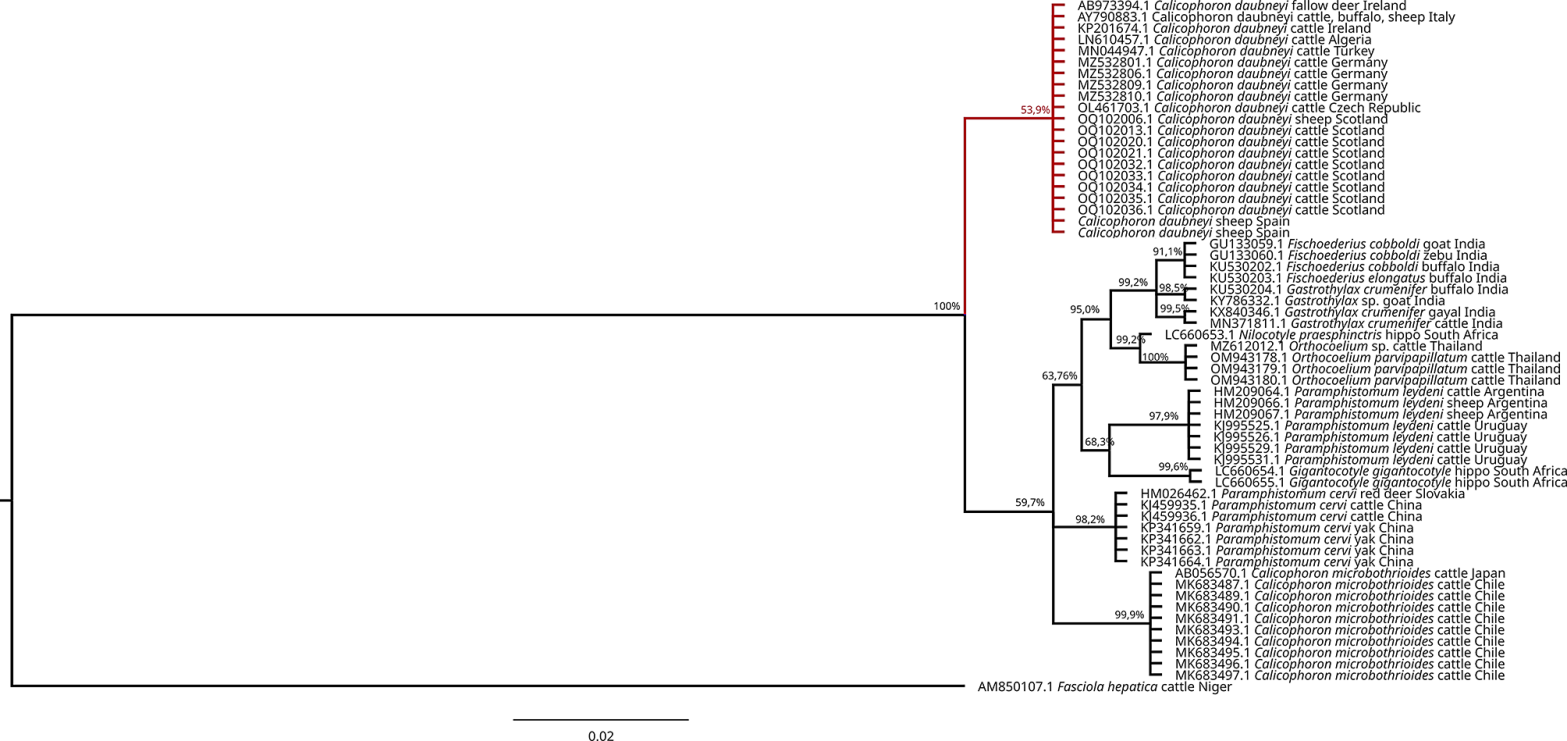
Phylogenetic tree of the sequences obtained from the ITS-2 region. Sequences corresponding to *Calicophoron daubneyi* in different host species and countries of Europe and the Mediterranean area are marked in red

Amplicons of the expected size were detected in six of the seven processed samples using the newly described primers. All the sequences were identical to the *C. daubneyi* sequences deposited in GenBank (Supplementary Table 3), and the phylogenetic tree grouped our sequences into a well-defined branch together with other *C. daubneyi* sequences obtained from domestic and wild ruminants from other European and Mediterranean countries (Fig. 4).

## Discussion

Considering the growing interest in paramphistomid infections in Europe in recent decades and their clinical importance in small ruminants [4], unravelling the current situation in sheep from areas where an increase in prevalence has been observed in cattle, one of the main definitive hosts, becomes essential. In this context, our results reveal that paramphistomids are not very prevalent (14%) in sheep from northwestern Spain but are widespread in all geoclimatic areas since positive animals were found in 44% of the flocks. Our data confirm a noticeable rise and expansion of rumen flukes in sheep from this region during the last 15 years, as available in previous information, with an individual prevalence under 1% [31] and flock-level prevalence ranging from 1.1 to 8.5% [31, 46, 47]; this trend is quite similar to that observed in cattle from the same region, where prevalence remained relatively stable (12–17%) between 2001 and 2005 [25, 48], increasing to 26% in 2010 [26]. In fact, the percentage of positive sheep found in the present study is similar to the prevalence reported in Galician cattle (10.1–13%) between 2001 and 2004 [23, 49].

The individual prevalence values reported in the present study are consistent with those reported in sheep from other Western European countries, such as the UK (13.5%) [18] or Ireland (14%) [50]; it is worth noting that other studies performed in the latter country reported prevalences of up to 50%, although with significant seasonal variations [13, 21]. In contrast, the percentages of positive sheep recorded in other European countries further East, such as Italy [14] and Germany [51], were much lower and did not exceed 4%. This situation agrees with that observed in cattle; thus, a thorough analysis of available data revealed the highest prevalence in the British Islands (25-53.8%) [16, 18, 52, 53], Spain (13-38.7%) [1, 26, 28, 54] and France (29.9–50%) [55, 56]. In contrast, rumen flukes are less common in other Central, Eastern and Southern European countries, such as Belgium [11], the Netherlands [15], Germany [51], the Czech Republic [12], Italy [16] and Greece [57], since the prevalence never reached 30%. These noticeable differences could be related to climatic conditions; those European regions where the highest prevalences were recorded show an oceanic climate characterized by moderate temperatures and abundant rainfall throughout the year [58], which favours the development of the external cycle of paramphistomids [59, 60]. Our results are also consistent with previous data demonstrating that the prevalence in cattle is, in general, much higher than that reported in sheep. In this context, small ruminants are considered less suitable hosts for the parasite than cattle, which represents the major definitive host [61, 62].

The absence of infections with adult paramphistomids in roe deer from the study area agrees with previous findings in the same region [1]. In fact, paramphistomid infections by *Paramphistomum microbothrium* and *Calicophoron daubneyi* in roe deer have been reported only in Eastern European countries such as Serbia (53%) [63] and Romania (7.7%) [34], respectively. These discrepancies may be due to differences in the management of domestic ruminants or in the habitat of wild ruminants between countries. The presence of the parasite in roe deer from other countries highlights the need to continue monitoring the situation in wild ruminants in the area.

The risk factor analysis allowed the detection of four variables significantly affecting the prevalence of infection. The probability of being infected with paramphistomids significantly increased with age since the longer the animals are exposed to the parasite on pastures, the greater the risk of becoming infected, as previously reported in cattle [1, 29]. The significant positive relationship between prevalence and age suggests that, after exposure to this trematode, no protective immunity is developed; previous studies have also demonstrated that paramphistomids, like other trematodes, secrete immunomodulatory proteins, leading to a favourable immune environment for the parasite [64, 65]. In contrast, other studies including sheep [66] and cattle [23, 67] have reported the highest prevalence in young animals; in this regard, the prevalence in young animals can be underestimated if coprological tests are performed during the 2–3-month prepatent period [17, 61, 62]. Thus, further research is needed to elucidate the role of age and immunity in the establishment of paramphistomid infections in sheep.

The higher probability of infection in sheep from the central climatic zone correlates well with previous results in cattle from the same study area [1]. The central area of Galicia has moderate temperatures and high rainfall, and the mean slope is lower than that in other areas [68]. These climatic and orographic characteristics can favour the external stages of the parasite, creating optimal habitats for its main intermediate host in Europe, *Galba truncatula*, as previously reported [69]. Furthermore, this area concentrates most livestock farms in the region, especially cattle. As cattle constitute the major reservoir of paramphistomids [1, 26] and are responsible for environmental contamination in this area, it is reasonable to find a relatively high probability of infection in sheep.

The logistic regression results also revealed that the absence of watering points on pastures significantly increased the risk of infection with paramphistomids. Pastures in this region often have wet areas providing suitable habitats for intermediate hosts [4, 69], where contamination with metacercariae is usually greater. For these reasons, the installation of water points away from wet areas prevents animals from drinking in and grazing around these areas, decreasing the risk of infection. It is worth noting that the presence of water troughs was considered a risk factor in arid areas, as the only wet areas of the pasture will be around them, and animals will also tend to graze there [70].

Finally, sheep from farms that use their own manure for fertilising pastures had the highest prevalence. Since all sheep included in this study went to grazing areas daily, this practice is expected to lead to a progressive increase in pasture contamination, especially if fresh nonfermented manure containing viable parasitic forms is used. In contrast, those farms employing external manure for fertilising fields usually use cattle manure from slurry pits, previously subjected to a fermentation process that destroys the parasitic forms, thus reducing the risk of contamination of the pasture with rumen fluke eggs [71]. Finally, manure from other nonruminant farms will not contribute to an increase in the parasite load of the pasture, as paramphistomid species identified in Europe are exclusive parasites of ruminants [4].

In addition, three factors significantly affected egg shedding intensity. Egg output increased with age until 72 months of age, and in general, these results are consistent with the prevalence findings. Thus, the absence of acquired protective immunity after reinfection together with the long lifespan of adult flukes [62, 72] leads to an accumulation of adult paramphistomids in the rumen, resulting in increasing egg counts with age.

A surprising finding was that egg shedding was lower on extensive farms than on semiextensive farms. However, the extensive farms included in the study were mainly small flocks with large grazing areas; in contrast, semiextensive flocks generally include a larger number of sheep grazing close to the facilities where they were kept at night. Thus, the higher stocking density on semiextensive farms could lead to a greater contamination in pastures, favouring reinfection and higher egg counts.

Those animals infected with *F. hepatica* excreted significantly greater paramphistomid egg counts than did the negative animals. Since both trematodes share the same intermediate host (*G. truncatula*) in Europe, those areas representing a risk for infection with both parasites are the same. Coinfections in intermediate hosts are uncommon since they compromise the survival of the snail [73–75]. In contrast, coinfections in the definitive host are frequent [15, 52, 76–78]. Notably, *F. hepatica* has been shown to secrete immunomodulatory products that negatively influence the host immune system, which may increase the excretion of eggs from other parasites [79].

This study provides the first molecular identification of paramphistomid species infecting sheep from Spain, being *C. daubneyi* the only species detected. Although our results agree which those previously reported in domestic ruminants from Spain [1, 29], as well as in most European countries [18, 35, 80], *P. leydeni* has occasionally been found in other wild and domestic ruminants from this continent [21, 22, 34, 35]. In this regard, it is worth noting that concurrent infections by other paramphistomid species may not be excluded in either our study or most of the molecular studies mentioned above since the PCR techniques used selectively amplify the dominant populations. For this reason, further molecular studies using species-specific primers are needed for a more robust characterisation of the paramphistomid population in this region.

## Conclusions

The present study revealed that *C. daubneyi* is the most common paramphistomid species in sheep flocks from northwestern Spain. Although its presence is currently moderate in sheep from this region, comparison with previous data clearly reveals an increasing trend in prevalence. For these reasons, continuing monitoring the status of rumen fluke infections in sheep, especially in areas with a high density of livestock, is strongly recommended. In this context, our results also demonstrate that special attention should be given to adult animals since they are the main carriers responsible for environmental contamination. In addition, detecting risk areas and applying effective control management measures such as the installation of watering points on pastures also seem essential for limiting infections in livestock, especially in sheep, since they are susceptible to developing clinical paramphistomidosis at any time in their lives.

## Electronic supplementary material

Below is the link to the electronic supplementary material.

Supplementary Material 1: Supplementary Table 1 *(.docx)*: DNA sequences encoding the ITS-2 region of Trematoda used for designing the novel primers for identifying paramphistomids

Supplementary Material 2: Supplementary Table 2 *(.docx)*: Primers used for the detection and identification of Trematoda species

Supplementary Material 3: Supplementary Table 3*(.docx)*: Sequence data of *Calicophoron daubneyi* isolates at the ITS-2 *region*, the closest reference sequences deposited in GenBank and the isolation source, country and percentage of identity of the deposited sequences

Supplementary Material 4: Supplementary Fig. 1*(.png)*: (A) Farm from the Coastal area, situated from sea level to 200 m, mean slope of 13–25%, with moderate precipitations and temperatures. Many areas of natural forests were replaced by non-autochthonous *Eucalyptus* sp. (B) Farm from the Central area at 200–650 m above sea level and low mean slope (< 13%), with low precipitations and moderate temperatures. Large autochthonous forest areas, with *Quercus robur* and *Castanea sativa*, are common. (C) Farm from Mountain area situated at 650–1285 m with high mean slope (> 25%), with low temperatures and high precipitations. Forests are composed of autochthonous tree species and coniferous trees. (D) Example of water troughs placed in the pastures

## Data Availability

The data supporting the conclusions of this article are included within the article. A more detailed dataset used during the current study is available from the corresponding author on reasonable request.

## Abbreviations

AIC: Akaike information criterion
BLAST: Basic Local Alignment Search Tool
epg: Egg per gram of faeces
HKY + G: Hasegawa-Kishino-Yano substitution model with gamma-distributed rate variation across sites
ITS-2: Internal transcribed spacer 2
opg: Oocysts per gram of faeces
SD: Standard deviation
