# Supplementary material for: Prevalence, risk factors and molecular identification of paramphistomid species in sheep from a Spanish endemic area
Source: Ir Vet J. 2024 Nov 26;77:21. doi: 10.1186/s13620-024-00283-y (PMC11590495; doi:10.1186/s13620-024-00283-y)
Supplement: Supplementary file 1 — Supplementary Material 1: Supplementary Table 1 (.docx): DNA sequences encoding the ITS-2 region of Trematoda used for designing the novel primers for identifying paramphistomids [file 13620_2024_283_MOESM1_ESM.docx]

Supplementary Table 1: DNA sequences encoding the ITS-2 region of Trematoda used for designing the novel primers for identifying paramphistomids

| **Genus** | **GenBank accession numbers** |
| --- | --- |
| *Calicophoron* spp. | AB973394.1; KP201674.1; MZ532801.1; MZ532806.1; MZ532810.1; MZ532809.1; |
| *Paramphistomum* spp. | KJ995526.1; KJ995529.1; HM209064.1; HM026462.1; KP341659.1; KP341664.1 |
| *Dicrocoelium* spp. | HM026461.1 |
| *Fasciola* spp. | OQ513950.1; ON661091.1; OQ513944.1 |
| *Schistosoma* spp. | OQ459348.1; OQ459347.1 |
| *Eurytrema* spp. | LC760844.1; LC760843.1 |
| *Haematoloechus* spp. | OQ354327.1; OQ354196.1 |
| *Diplodiscus* spp. | OP693657.1; OQ353091.1 |
