# Supplementary material for: Prevalence, risk factors and molecular identification of paramphistomid species in sheep from a Spanish endemic area
Source: Ir Vet J. 2024 Nov 26;77:21. doi: 10.1186/s13620-024-00283-y (PMC11590495; doi:10.1186/s13620-024-00283-y)
Supplement: Supplementary file 2 — Supplementary Material 2: Supplementary Table 2 (.docx): Primers used for the detection and identification of Trematoda species [file 13620_2024_283_MOESM2_ESM.docx]

Supplementary Table 2: Primers used for the detection and identification of Trematoda species

| **Target region** | **Name** | **Primer sequence 5’- 3’** | **Fragment size** | **nt*** | **Tm °C** | **CG%** | **Self-dimers** | **Cross primer dimers** |
| --- | --- | --- | --- | --- | --- | --- | --- | --- |
| ***ITS-2*** | ITS2_TremF | ACTGCATACTGCTTTGAACAT | 400 bp | 21 | 59.4°C | 38.1 | None | None |
|  | ITS2_TremR | AAGTTCAGCGGGTATTCACG |  | 20 | 64.3°C | 50 | None | None |
