# Supplementary material for: Prevalence, risk factors and molecular identification of paramphistomid species in sheep from a Spanish endemic area
Source: Ir Vet J. 2024 Nov 26;77:21. doi: 10.1186/s13620-024-00283-y (PMC11590495; doi:10.1186/s13620-024-00283-y)
Supplement: Supplementary file 3 — Supplementary Material 3: Supplementary Table 3(.docx): Sequence data of Calicophoron daubneyi isolates at the ITS-2 region, the closest reference sequences deposited in GenBank and the isolation source, country and percentage of identity of the deposited sequences [file 13620_2024_283_MOESM3_ESM.docx]

Supplementary Table 3: Sequence data of Calicophoron daubneyi isolates at the ITS-2 gene, the closest reference sequences deposited in GenBank and the isolation source, country and percentage of identity of the deposited sequences.

| **Reference sequence** | **Isolation source** | **Country** | **Identities (bp)** | **Identity (%)** | **Reference** |
| --- | --- | --- | --- | --- | --- |
| OQ102006.1-OQ102007.1; OQ102009.1- OQ102010.1; OQ102015.1; OQ102017.1- OQ102021.1; OQ102023.1; OQ102025.1; OQ102031.1; OQ102033.1-OQ102034.1; OQ102036.1; OQ102037.1 | Cattle | Scotland | 403/403 | 100 | Busin et al., 2023 |
| OQ102006.1-OQ102007.1; OQ102009.1-OQ102010.1 | Sheep |  |  |  | Busin et al., 2023 |
| KP201674 | Cattle | Ireland | 403/403 | 100 | Chryssafidis et al., 2015 |
| LN610458.1 | Cattle, Buffalo, Sheep | Italy | 403/403 | 100 | Rinaldi et al., 2005 |
| OQ102008.1 | Sheep | Scotland | 403/404 | 99.75 | Busin et al., 2023 |
| OQ102024.1; OQ102029.1-OQ102030.1; OQ102032.1; OQ102035.1 | Cattle |  |  |  |  |
| OQ102026.1-OQ102027.1 | Cattle | Scotland | 403/405 | 99.51 | Busin et al., 2023 |
| OQ102011.1 | Sheep | Scotland | 400/402 | 99.50 | Busin et al., 2023 |
| AB973394.1 | Fallow deer | Ireland | 401/403 | 99.50 | Naranjo-Lucena et al., 2018 |
