# Supplementary figures and images for: Prevalence, risk factors and molecular identification of paramphistomid species in sheep from a Spanish endemic area
Source: Ir Vet J. 2024 Nov 26;77:21. doi: 10.1186/s13620-024-00283-y (PMC11590495; doi:10.1186/s13620-024-00283-y)

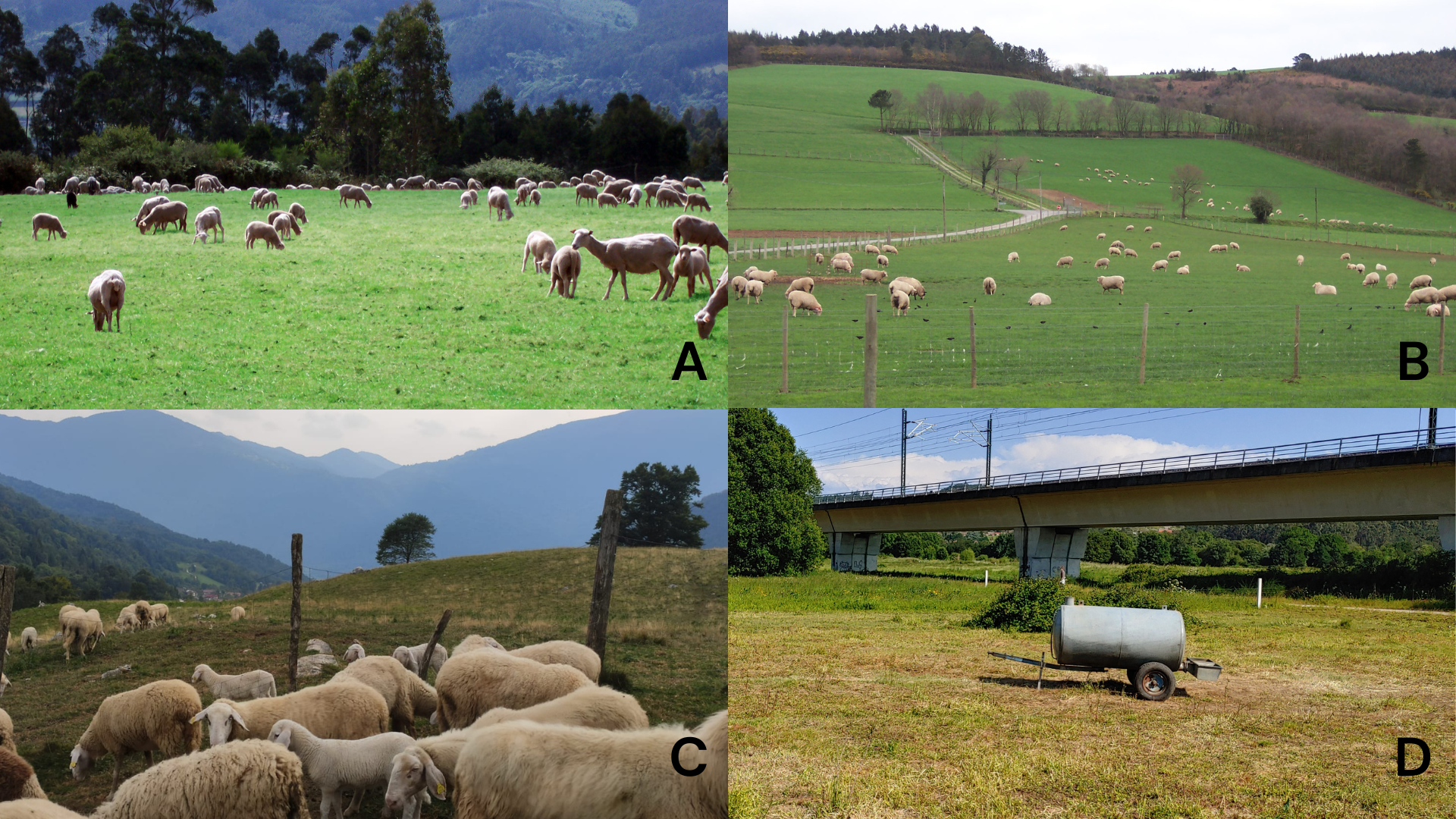

Supplement: Supplementary file 4 — Supplementary Material 4: Supplementary Fig. 1(.png): (A) Farm from the Coastal area, situated from sea level to 200 m, mean slope of 13–25%, with moderate precipitations and temperatures. Many areas of natural forests were replaced by non-autochthonous Eucalyptus sp. (B) Farm from the Central area at 200–650 m above sea level and low mean slope (< 13%), with low precipitations and moderate temperatures. Large autochthonous forest areas, with Quercus robur and Castanea sativa, are common. (C) Farm from Mountain area situated at 650–1285 m with high mean slope (> 25%), with low temperatures and high precipitations. Forests are composed of autochthonous tree species and coniferous trees. (D) Example of water troughs placed in the pastures [file 13620_2024_283_MOESM4_ESM.png]
